# Supplementary material for: Transparent radiative cooling cover window for flexible and foldable electronic displays
Source: Nat Commun. 2024 May 24;15:4443. doi: 10.1038/s41467-024-48840-x (PMC11126687; doi:10.1038/s41467-024-48840-x)
Supplement: Supplementary file 1 — Supplementary Information [file 41467_2024_48840_MOESM1_ESM.pdf]

Supplementary Information

Transparent radiative cooling cover window for flexible and foldable electronic displays

Kang Won Lee[1†], Jonghun Yi[1†], Min Ku Kim[1], Dong Rip Kim[1*]

[1]School of Mechanical Engineering, Hanyang University, Seoul 04763, South Korea

* Corresponding author. E-mail: dongrip@hanyang.ac.kr

[†] These authors contributed equally to this work.

This PDF file includes:

Supplementary Figure : 1 ~ 15
Supplementary Table : 1 ~ 5
Supplementary References : 1 ~ 52

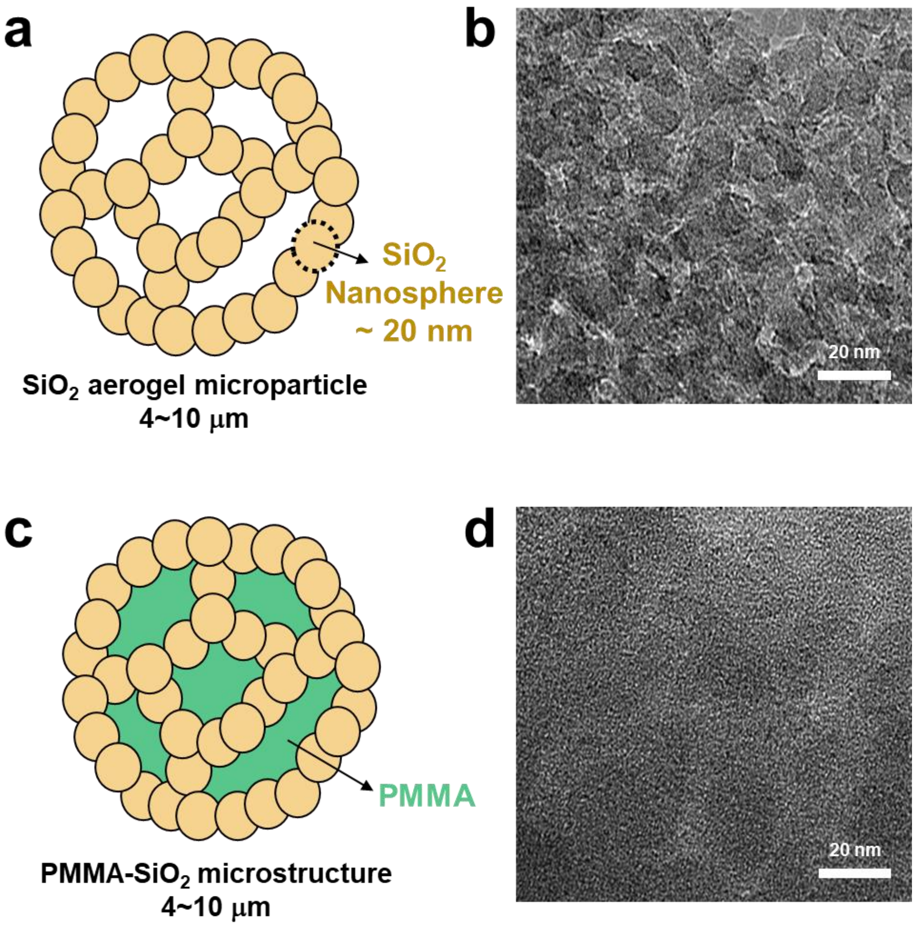

Supplementary Figure 1. a, Schematic to illustrate a SiO_2 aerogel microparticle. **b,** Transmission Electron Microscopy (TEM) image to show SiO_2 aerogel microparticles. **c,** Schematic to illustrate a PMMA-SiO_2 microstructure. **d,** TEM image to show PMMA-SiO_2 microstructures. Figure **a** and **c** were reproduced with permission from Elsevier[1].

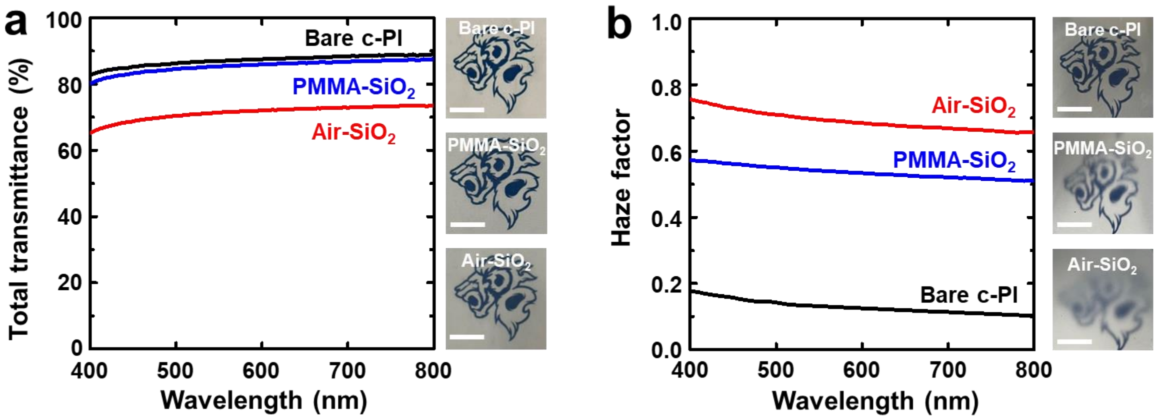

Supplementary Figure 2. Optical characteristics of metamaterial (24 wt% PMMA-SiO_2 microstructures), 24 wt% air-SiO_2 microstructures in c-PI, and bare c-PI in visible wavelengths. a, Total transmittance and **b,** haze factor in visible wavelengths (400-800 nm). All the samples have 50 μm thickness. Scale bar is 2 cm. Source data are provided as a Source Data file.

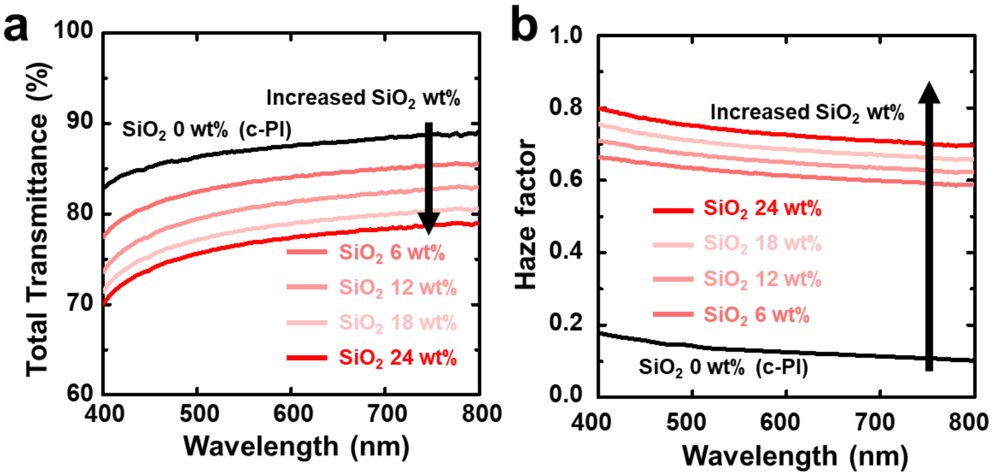

Supplementary Figure 3. Optical characteristics of the air-SiO_2 microstructures in c-PI in terms of air-SiO_2 microstructures contents (0-24 wt%) in visible wavelengths. a, Total transmittance and **b,** haze factor in visible wavelengths (400-800 nm). All the samples have 50 μm thickness. Source data are provided as a Source Data file.

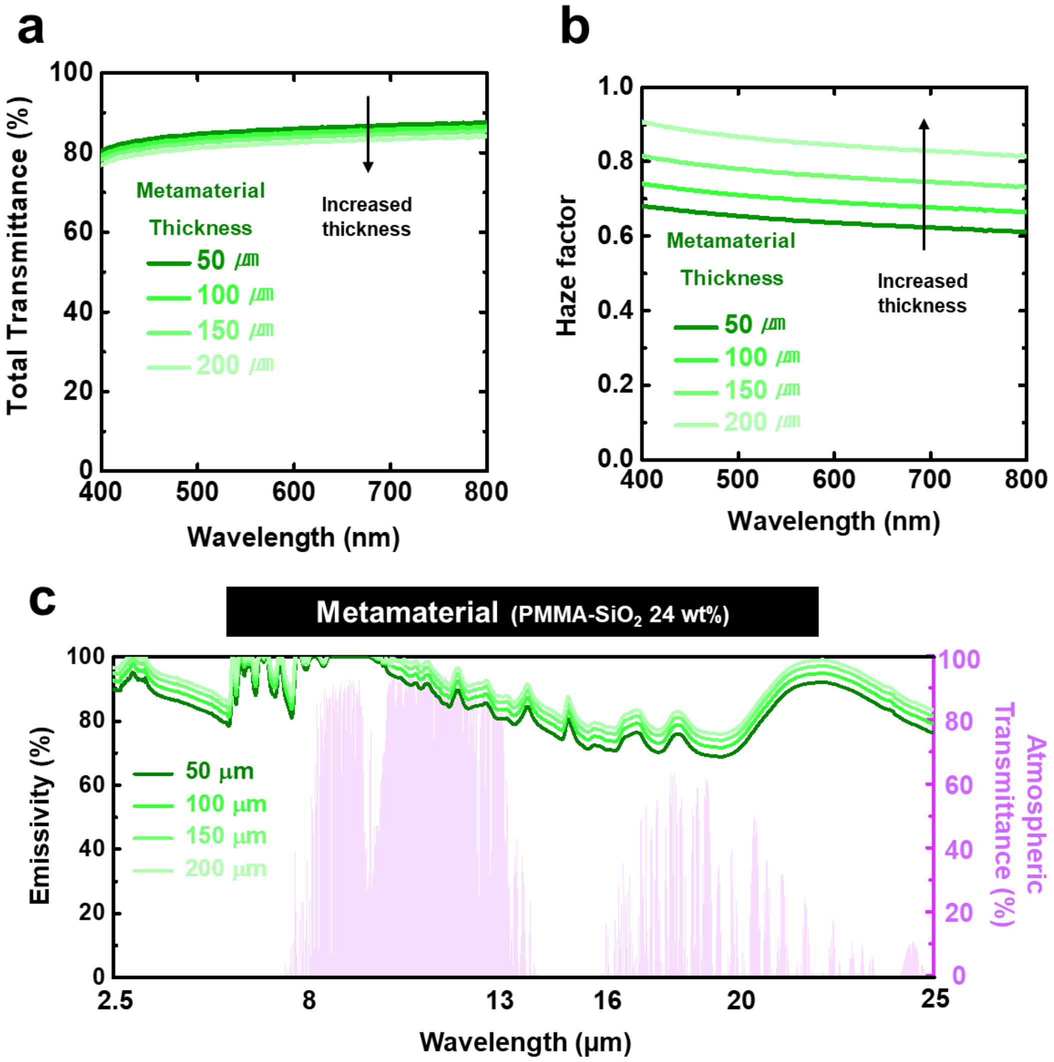

Supplementary Figure 4. Measured optical properties of metamaterials (24 wt% PMMA-SiO_2 microstructures) with different thicknesses. **a,** Light transmission at wavelengths of 400-800 nm. The total transmittance of metamaterials is 85.5%, 84.5%, 83.3%, and 82.0% for thicknesses of 50, 100, 150, and 200 μm, respectively. **b,** Haze factor at wavelengths of 400-800 nm. The haze factor of metamaterials is 0.64, 0.70, 0.77, and 0.85 for thicknesses of 50, 100, 150, and 200 μm, respectively. **c,** Emissivity in the atmospheric window (wavelengths of 8-13 μm). The integrated emissivity of metamaterials in the atmospheric window is 94.6, 95.3, 95.7, and 95.9% for thicknesses of 50, 100, 150, and 200 μm, respectively. Source data are provided as a Source Data file.

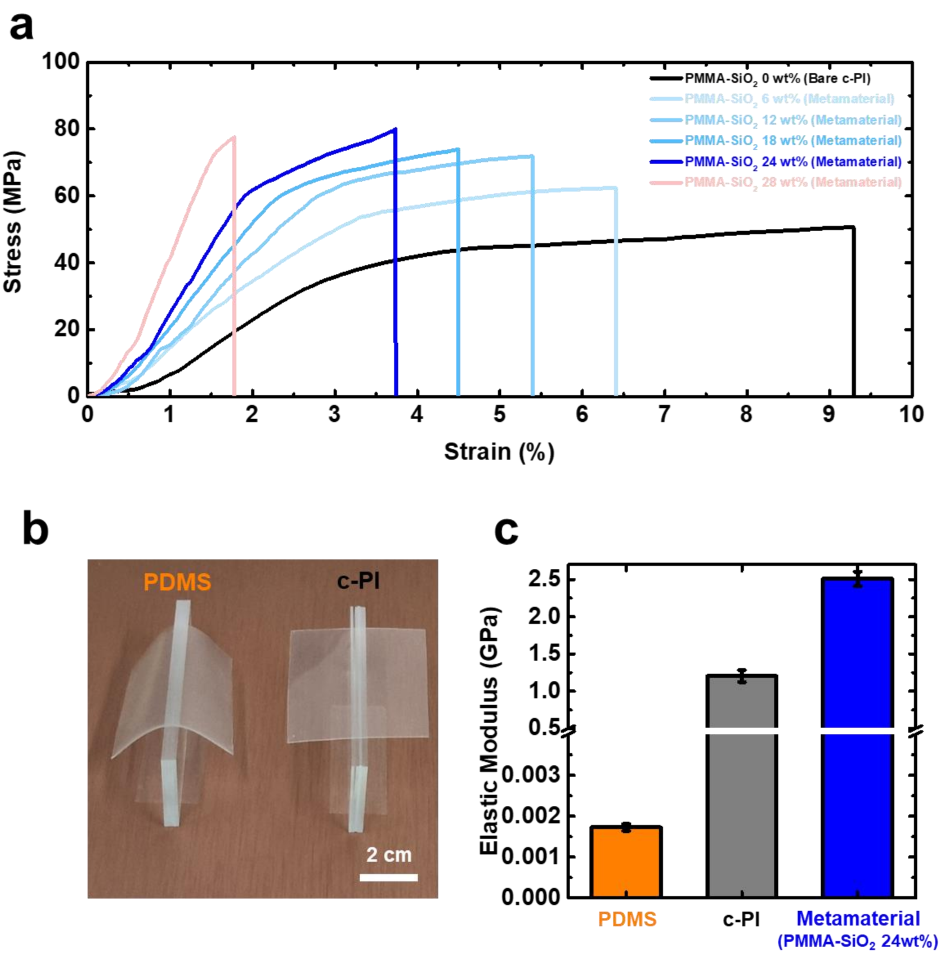

Supplementary Figure 5. Mechanical properties of metamaterials with a film thickness of 50 μm. a, Engineering stress-strain curves according to the contents of PMMA-SiO_2 microstructures in the metamaterial. **b,** Photograph to show the flat and stiff condition of c-PI as cover windows of foldable and flexible displays, compared to PDMS. **c,** Elastic modulus of PDMS, c-PI, and metamaterial (24 wt% PMMA-SiO_2 microstructures). Error bars in **c** indicate variations in measurements of the samples, displaying the mean and standard distribution (n=15). Source data are provided as a Source Data file.

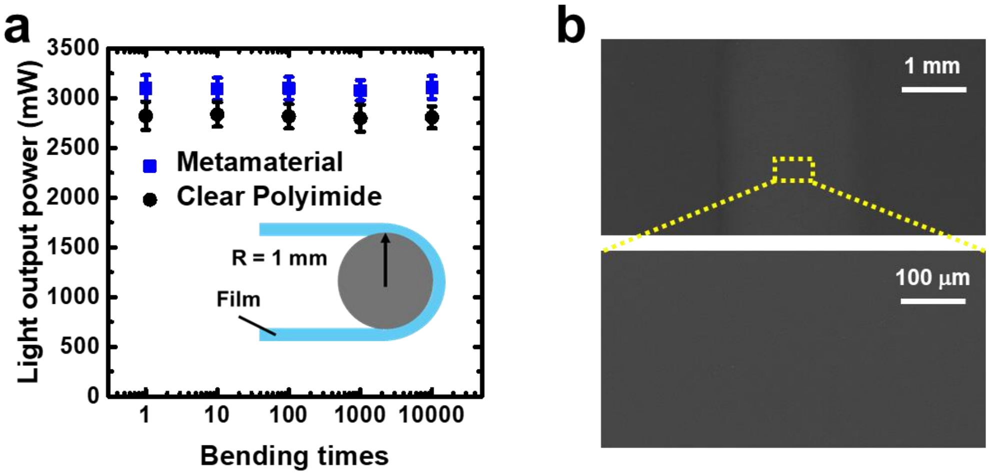

Supplementary Figure 6. Bending stability test results of metamaterials (24 wt% PMMA-SiO_2 microstructures) under repeated folding and releasing process. a, Light output power changes of the LED chip on which c-PI or metamaterial films are placed in terms of bending cycles. The inset schematic illustrates the experimental setup of bending test with a bending radius of 1 mm. **b,** SEM images of the metamaterial film after 10,000 bending cycles. Tested samples have the thickness of 50 μm. Error bars in **a** indicate variations in measurements of the samples, displaying the mean and standard distribution (n=30). Source data are provided as a Source Data file.

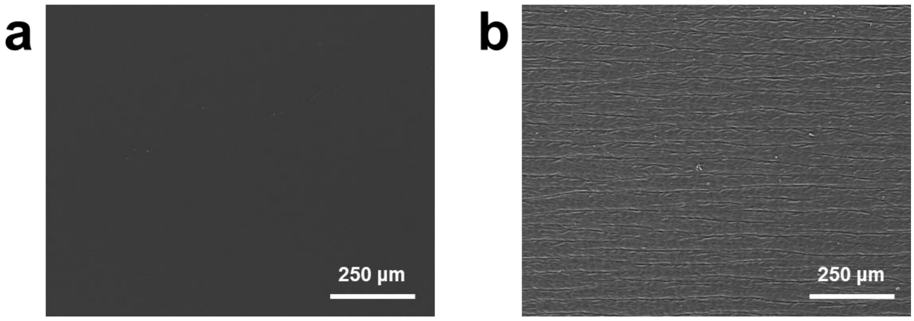

Supplementary Figure 7. Scanning Electron Microscopy (SEM) images after bending cycle test with a bending radius of 1 mm for 10,000 bending times. a, metamaterial (24 wt% PMMA-SiO_2 microstructures in c-PI). **b,** PDMS. The sample thicknesses were identical as 50 μm.

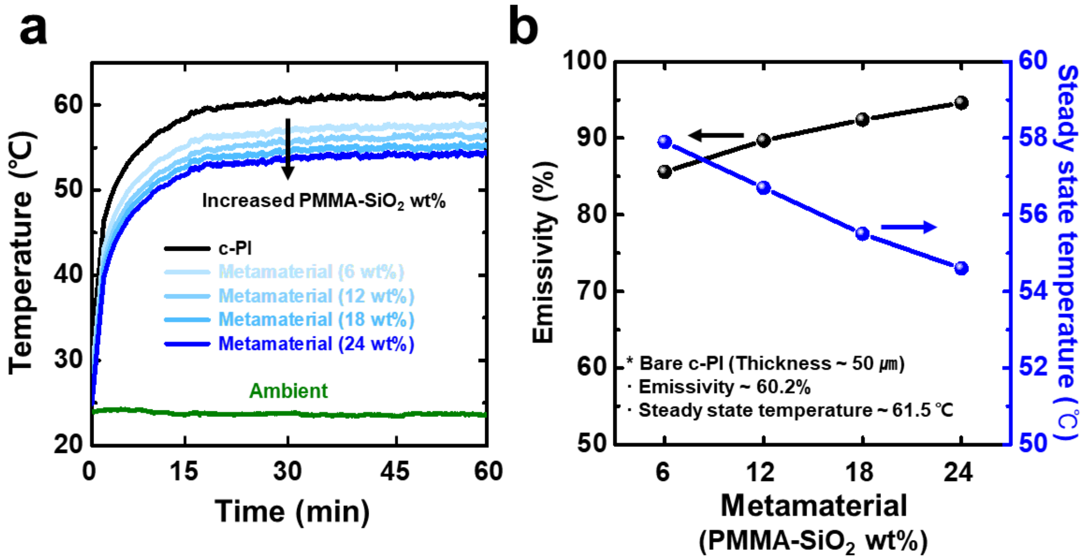

Supplementary Figure 8. Measured cooling characteristics of 50 μm thick metamaterial films in terms of the contents of PMMA-SiO_2 microstructures under AM 1.5G solar illumination (1,000 W/m^2) in indoor condition. a, Surface temperatures of the simulated display (heat generation of 100 W/m^2) by equipping the metamaterial films (0~24 wt% PMMA-SiO_2 microstructures in c-PI) on top of the simulated displays. **b,** Emissivity in the atmospheric window of 50 μm thick metamaterial films (6~24 wt% PMMA-SiO_2 microstructures in c-PI) and steady-state temperature of the simulated display surface with heat generation of 100 W/m^2 on which the metamaterial film was placed. Source data are provided as a Source Data file.

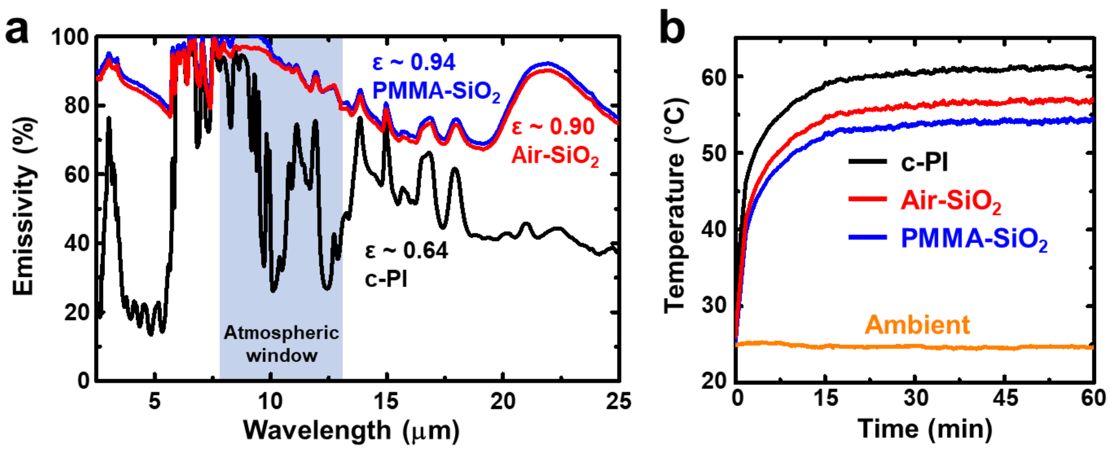

Supplementary Figure 9. a, Emissivity in the atmospheric window for metamaterial (24 wt% PMMA-SiO_2 microstructures), 24 wt% air-SiO_2 microstructures in c-PI, and bare c-PI. All the samples have an identical thickness of 50 μm. **b,** Radiative cooling performance of metamaterial (24 wt% PMMA-SiO_2 microstructures), 24 wt% air-SiO_2 microstructures in c-PI, and bare c-PI placed on top of the simulated display with heat generation of 100 W/m^2 in indoor conditions. Source data are provided as a Source Data file.

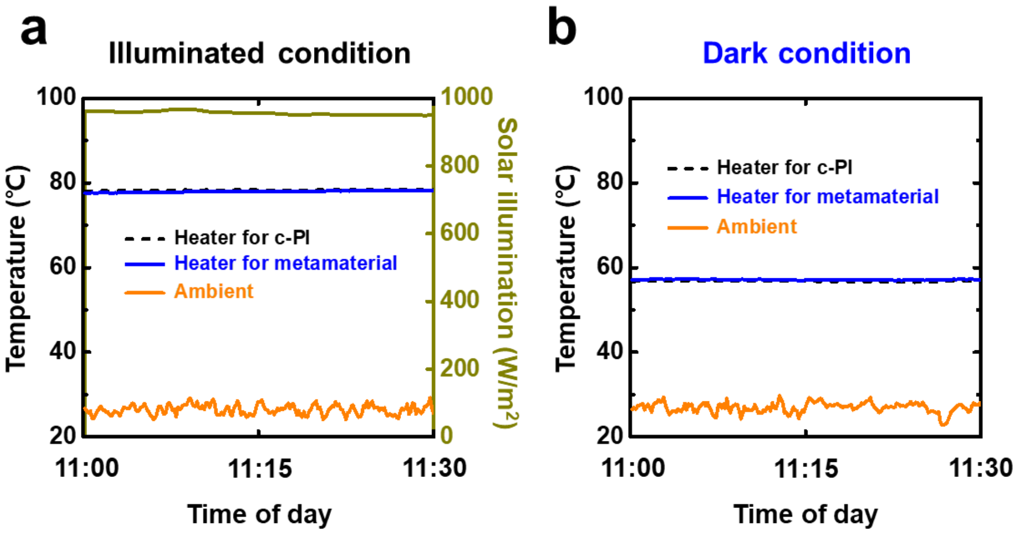

Supplementary Figure 10. Measured temperatures of simulated display heaters without being covered by the samples under illuminated and dark conditions. Source data are provided as a Source Data file.

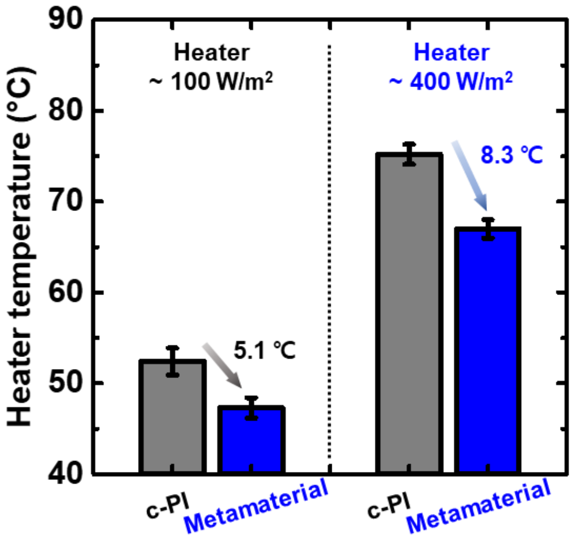

Supplementary Figure 11. Measured steady-state temperatures of samples on the front of heaters with different power densities in outdoor conditions (Seoul, South Korea) (100 W/m^2, 400 W/m^2). Error bars in indicate variations in measurements of the samples, displaying the mean and standard distribution (n=30). Source data are provided as a Source Data file.

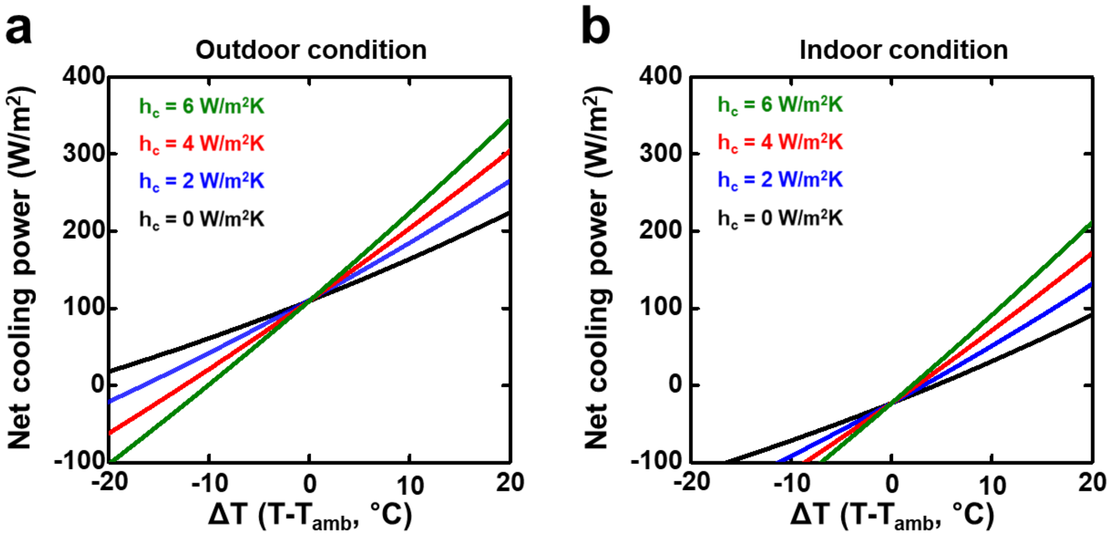

Supplementary Figure 12. Net cooling power of transparent radiative cooling metamaterials in terms of ΔT(=T-T_{amb}) and non-radiative heat exchange coefficients (h_c) for a, the outdoor and b, the indoor conditions. Source data are provided as a Source Data file.

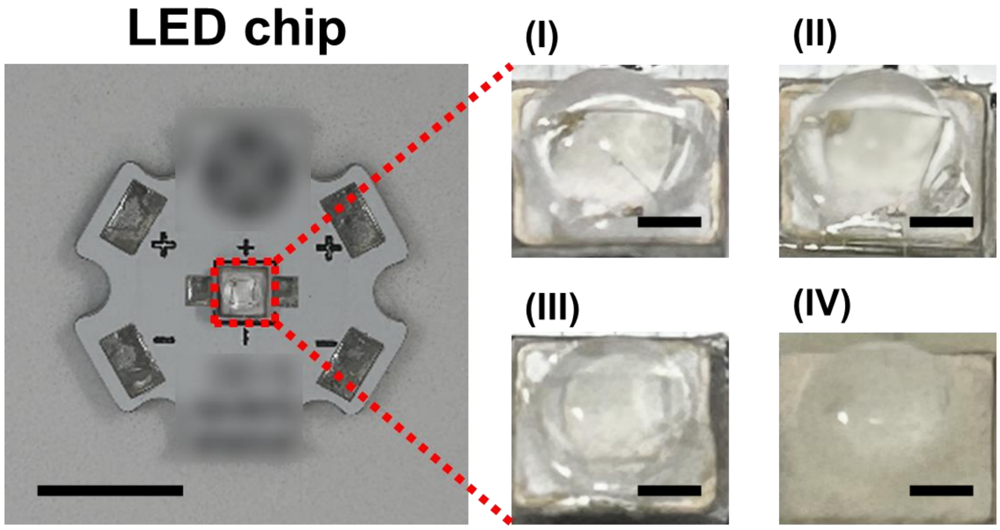

Supplementary Figure 13. Optical images of (Left) the LED chip covered with the silicone lens and (Right) the 50 μm thick metamaterial film integrated on top of the LED chip covered by the silicone lens. (I) bare LED chip covered by the silicone lens, (II) the film attachment with a full adhesive layer (c-PI), (III) the integration by direct coating of the mixture solution, and (IV) the film placement with the air void between the film and the device as a control sample. (Left) scale bar is 1 cm. (Right) All the scale bars are 1 mm.

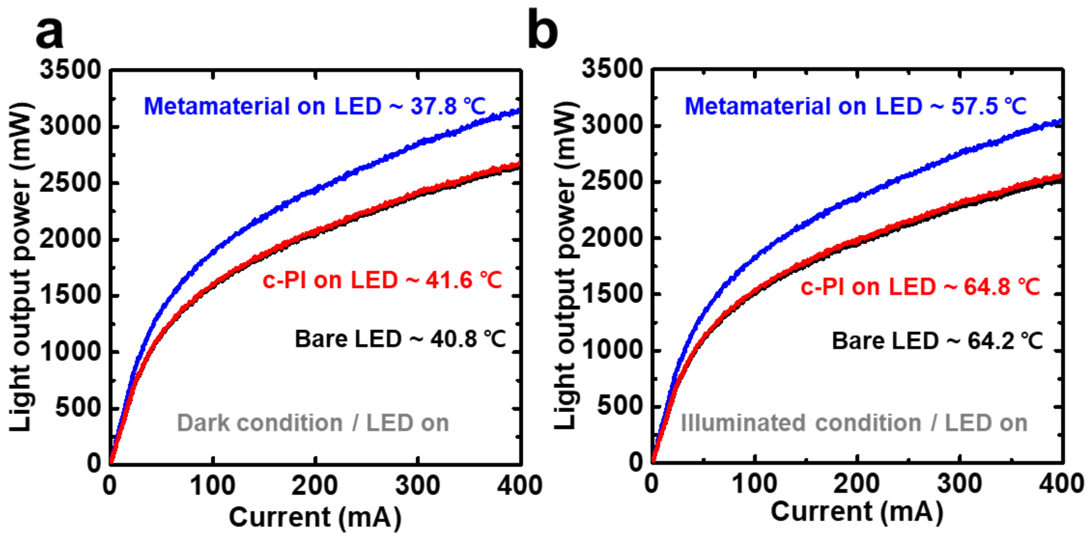

Supplementary Figure 14. Light output power as a function of injection current for the LEDs with bare LED, c-PI on LED, and Metamatieral on LED under dark and illumination conditions. To simulate illuminated conditions, the light output pwer of LEDs was measured when the temperatures of the samples reached the steady-state temperatures of the samples under illuminated conditions. Source data are provided as a Source Data file.

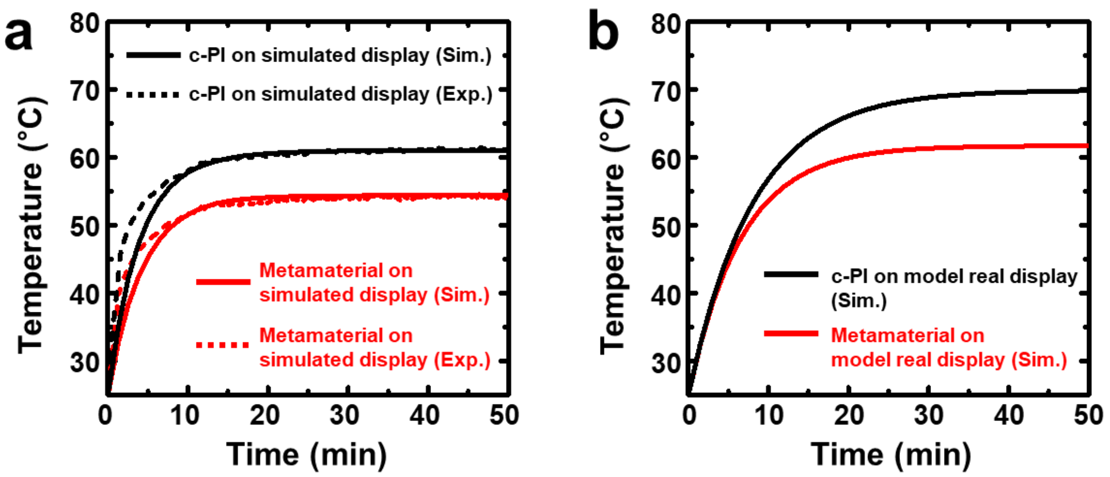

Supplementary Figure 15. **The estimated temperature responses of the simulated displays and the model real displays upon the integration of the metamaterials under illuminated condition in indoor environments. a,** Temperature responses of the simulated displays with c-PI and metamaterial. The estimated temperature responses of the simulated displays agree well with the experimental results. **b,** Estimated temperature responses of the model real displays with c-PI and metamaterial. Source data are provided as a Source Data file.

Supplementary Table 1. Comparison of the recent studies on the transparent radiative cooling materials.

Material	Transmission in visible wavelengths	Emissivity in atmospheric window	Solar intensity / Relative humidity	Temperature drop (control sample)	Elastic modulus	WVTR	Reference
SiO_2 microspheres in TPX	-	>0.93	~900 W/m^2 / -	-	~360 MPa* (TPX)	~775 g/m^2·day† (TPX)	2
SiO_2 photonic crystal arrays	-	>0.90	~1000 W/m^2 / -	13 °C (Si wafer)	130~188 GPa‡ (SiO_2)	~2·10^{-3} g/m^2·day§ (SiO_2)	3
SiO_2 microspheres on glass	~84%	>0.98	800 W/m^2 / ~37%	14 °C (Si wafer)	130~188 GPa‡ (SiO_2)	~2·10^{-3} g/m^2·day§ (SiO_2)	4
Grating SiO_2 structure	~90%	~0.90	830~990 W/m^2 /30~40%	3.6 °C (Si wafer)	130~188 GPa‡ (SiO_2)	~2·10^{-3} g/m^2·day§ (SiO_2)	5
SiO_2/TiO_2 multi-layer on ITO-coated PET film	~87%	~0.88	~700 W/m^2 / -	9.6 °C (Roof)	130~188 GPa‡ (SiO_2)	~2·10^{-3} g/m^2·day§ (SiO_2)	6
Silk fibroin	~91%	~0.88	~900 W/m^2 / -	5.1 °C (Solar panel)	~1 GPa** (Silk fibroin)	2,000 g/m^2·day†† (Silk fibroin)	7
PDMS on ITO-coated window	~94%	~0.90	~630 W/m^2 / ~13%	7 °C (Bare window)	~2.61 MPa‡‡ (PDMS)	708 g/m^2·day§§ (PDMS)	8
n-hexadecane-infiltrated SiO_2 aerogel microparticles in PDMS	~91%	~0.98	~920 W/m^2 / 30~45%	7.7 °C (Solar cell)	~2.61 MPa‡‡ (PDMS)	708 g/m^2·day§§ (PDMS)	9
Microstructured PDMS	>90%	Close to 1.0	-	4 °C (Solar cell)	~2.61 MPa‡‡ (PDMS)	708 g/m^2·day§§ (PDMS)	10
Nanofibrillated cellulose film	>90%	>0.85	~800 W/m^2 / ~27%	5 °C (Si wafer)	46 GPa*** (Cellulose)	1,315 g/m^2·day††† (Cellulose)	11
**PMMA-SiO_2 microstructures in c-PI**	**~85%**	**~0.95**	**~850 W/m^2 / 30~40%**	**8.3 °C (c-PI on simulated display (400W/m^2))**	**2.51 GPa**	**66 g/m^2·day**	**Our work**

* Elastic modulus of TPX[12], † WVTR of TPX[13], ‡ Elastic modulus of SiO_2[14], § WVTR of SiO_2[15], ** Elastic modulus of silk fibroin[16], †† WVTR of silk fibroin[17], ‡‡ Elastic modulus of PDMS[18], §§ WVTR of PDMS[19], *** Elastic modulus of cellulose[20], ††† WVTR of cellulose[21]

Supplementary Table 2. Refractive index, surface energy, and solubility parameter of c-PI and PMMA. References of refractive indices[22,23], surface energy[24,25], and solubility parameters[26,27] are noted at the end of Supporting Information.

Material	n (refractive index) @ 632 nm	Surface energy (mN/m)	Solubility parameter (σ_t, (MPa)$^{1/2}$)
c-PI	1.50	40.0	22.6
PMMA	1.49	35.1	23.1

Supplementary Table 3. Comparison of pristine materials in terms of optical transparency, mechanical property (foldability at a bending radius of 1 mm), moisture impermeability, thermal stability, and touch sensitivity for the cover windows of foldable and flexible displays.

Pristine material	Optical transparency (Visible wavelengths)	Mechanical property (Foldability @ bending radius of 1 mm)	Moisture impermeability (g/m^2 day)	Touch sensitivity (Dielectric constant @ 1×10^6 Hz)	Thermal stability
Ultra-thin glass	Good (>90%)†	Weak* (Elastic modulus ~70 GPa, Tensile strength ~3 GPa)‡	Good (~0.002)§	Good (~3.9)**	Good (T_g = ~552 °C) ††
Clear polyimide	Good (~90%)†	Good (Elastic modulus 1~2 GPa, Tensile strength 60-100 MPa)‡	Good (~165)§	Good (3-4)**	Good (T_g > 400 °C) ††
PDMS	Good (~95%)†	Week (Elastic modulus 1.3~3.0 MPa, Tensile strength 3.5~7.7 MPa)‡	Weak (~708)§	Fair (~2.4)**	Weak (T_g = -127~-121 °C, Boiling point 155~200 °C) ††
PET	Good (>90%)†	Good (Elastic modulus ~3 GPa, Tensile strength ~80 MPa)‡	Good (~18)§	Fair (~2.2)**	Weak (T_g = 67~81 °C) ††
PVDF	Good (80-90%)†	Good (Elastic modulus 2.5-3.5 GPa, Tensile strength 40-60 MPa)‡	Good (~29)§	Weak (~0)**	Weak (T_g = -40 °C, Melting point 171~180 °C) ††
TPX	Good (>90%)†	Weak (Elastic modulus ~360 MPa, Tensile strength ~20 MPa)‡	Weak (~775)§	Fair (~2.0)**	Weak (T_g = 20 ~ 30 °C) ††
Cellulose	Good (85-90%)†	Good (Elastic modulus ~46 GPa, Tensile strength 2-3 GPa)‡	Weak (~1,315)§	Good (~3.0)**	Weak (T_g = ~107 °C) ††

* Foldability of ultra-thin glass (a thickness of ~ 85 μm) was reported at a bending radius of 3 mm[28].
† Optical transparency: Ultra-thin glass[29], Clear polyimide[30,31], PDMS[9], PET[32], PVDF[33], TPX[2], Cellulose[34]
‡ Elastic modulus, Tensile strength: Ultra-thin glass[28], Clear polyimide[30,31], PDMS[35], PET[36], PVDF[37], TPX[12], Cellulose[20]
§ Water vapor transmission rate: Ultra-thin glass[38], Clear polyimide[39], PDMS[19], PET[40], PVDF[41], TPX[13], Cellulose[21]
** Dielectric constant: Ultra-thin glass[42], Clear polyimide[30], PDMS[43], PET[44], PVDF[45], TPX[46], Cellulose[43]
†† Thermal stability: Ultra-thin glass[47], Clear polyimide[31], PDMS[48], PET[49], PVDF[50], TPX[51], Cellulose[52]

Supplementary Table 4. Performance of commercial LED with metamaterial in terms of integration methods. The thickness of all the metamaterial samples was ~50 μm.

Sample	Integration method	Light output power @ 350 mA	Steady-state temperature	
			Dark condition	Illuminated condition
Metamaterial on LED	Film attachment with an adhesive layer	3,106 mW	37.8 °C	57.5 °C
	Integration by direct coating	3,021 mW	36.2 °C	57.3 °C
	Film placement with the air void between the film and the device	2,549 mW	40.2 °C	59.1 °C
c-PI on LED	Film attachment with an adhesive layer	2,703 mW	41.6 °C	64.8 °C
LED	-	2,628 mW	40.8 °C	64.2 °C

Supplementary Table 5. The parameter values used in the simulation to estimate the temperature response of the simulated display and the model real display in indoor environments. It should be noted that P_{sun} is estimated under solar irradiation of AM 1.5G illumination, considering the light absorption of the displays between wavelengths of 300 nm to 2.5 μm. To estimate P_{surr} and $P_{non\text{-}rad}$, the surrounding emissivity (ε_{surr}) and the combined non-radiative heat transfer coefficient (h_c) was set as ~0.95 and ~5 W/m^2·K, respectively. P_{gen} was 100 W/m^2. The area of the simulated display and the model real display was set as 16 cm^2.

Sample	Mass (g)	Heat capacity (J/kg·K)	P_{sun} (W/m^2)	Emissivity (2.5~25 μm)
c-PI on simulated display	50	850	370	0.2~0.7
Metamaterial on simulated display	50	850	380	0.7~0.9
c-PI on model real display	40	1,500	335	0.2~0.7
Metamaterial on model real display	40	1,500	340	0.7~0.9

Supplementary References

1. Maleki H, *et al.* An overview on silica aerogels synthesis and different mechanical reinforcing strategies. *Journal of Non-Crystalline Solids* **385**, 55-74 (2014).
2. Zhai Y, *et al.* Scalable-manufactured randomized glass-polymer hybrid metamaterial for daytime radiative cooling. *Science* **355**, 1062-1066 (2017).
3. Zhu L, *et al.* Radiative cooling of solar absorbers using a visibly transparent photonic crystal thermal blackbody. *Proceedings of the National Academy of Sciences* **112**, 12282-12287 (2015).
4. Jaramillo-Fernandez J, *et al.* A Self-Assembled 2D Thermofunctional Material for Radiative Cooling. *Small* **15**, 1905290 (2019).
5. Zhao B, *et al.* Radiative cooling of solar cells with micro-grating photonic cooler. *Renewable Energy* **191**, 662-668 (2022).
6. Zhu Y, *et al.* Color-preserving passive radiative cooling for an actively temperature-regulated enclosure. *Light: Science & Applications* **11**, 122 (2022).
7. Chen Y-H, *et al.* Eco-Friendly Transparent Silk Fibroin Radiative Cooling Film for Thermal Management of Optoelectronics. *Advanced Functional Materials* **33**, 2301924 (2023).
8. Zhou Z, *et al.* Transparent Polymer Coatings for Energy-Efficient Daytime Window Cooling. *Cell Reports Physical Science* **1**, 100231 (2020).
9. Lee KW, *et al.* Visibly Clear Radiative Cooling Metamaterials for Enhanced Thermal Management in Solar Cells and Windows. *Advanced Functional Materials* **32**, 2105882 (2022).
10. Wang K, *et al.* Radiative cooling of commercial silicon solar cells using a pyramid-textured PDMS film. *Solar Energy* **225**, 245-251 (2021).
11. Gamage S, *et al.* Reflective and transparent cellulose-based passive radiative coolers. *Cellulose* **28**, 9383-9393 (2021).
12. Ilyin S, *et al.* Formation of Microfiltration Membranes from PMP/PIB Blends: Effect of PIB Molecular Weight on Membrane Properties. *Membranes* **10**, 9 (2020).
13. Wu H-Y, *et al.* Thin-Film Coated Plastic Wrap for Food Packaging. *Materials* **10**, 821 (2017).
14. Hopcroft MA, *et al.* What is the Young's Modulus of Silicon? *Journal of Microelectromechanical Systems* **19**, 229-238 (2010).
15. Lee WJ, *et al.* Environmental reliability and moisture barrier properties of silicon nitride and silicon oxide films using roll-to-roll plasma enhanced chemical vapor deposition. *Thin Solid Films* **720**, 138524 (2021).
16. Ling S, *et al.* Integration of Stiff Graphene and Tough Silk for the Design and Fabrication of Versatile Electronic Materials. *Advanced Functional Materials* **28**, 1705291 (2018).
17. Zhang X, *et al.* Fabrication and Characterization of Silk Fibroin/Curcumin Sustained-Release Film. *Materials* **12**, 3340 (2019).
18. Wang Z, *et al.* Crosslinking effect on polydimethylsiloxane elastic modulus measured by custom-built compression instrument. *Journal of Applied Polymer Science* **131**, 41050 (2014).
19. Kang D, *et al.* Study of the hybrid parylene/PDMS material. In: *2015 28th IEEE International Conference on Micro Electro Mechanical Systems (MEMS)*) (2015).
20. Guo W, *et al.* Processing bulk natural bamboo into a strong and flame-retardant composite material. *Industrial Crops and Products* **138**, 111478 (2019).
21. Song Z, *et al.* Hydrophobic-modified nano-cellulose fiber/PLA biodegradable composites for lowering water vapor transmission rate (WVTR) of paper. *Carbohydrate Polymers* **111**, 442-448 (2014).

22. Deka N, *et al.* Methyl Methacrylate-Based Copolymers: Recent Developments in the Areas of Transparent and Stretchable Active Matrices. *ACS Omega* **7**, 36929-36944 (2022).
23. Ando S, *et al.* Wavelength Dependence of Refractive Indices of Polyimides in Visible and Near-IR Regions. *Japanese Journal of Applied Physics* **41**, 5254-5258 (2002).
24. Hu G, *et al.* Functional inks and printing of two-dimensional materials. *Chem Soc Rev* **47**, 3265-3300 (2018).
25. Liu L, *et al.* Two-In-One Method for Graphene Transfer: Simplified Fabrication Process for Organic Light-Emitting Diodes. *ACS Appl Mater Interfaces* **10**, 7289-7295 (2018).
26. Bussi Y, *et al.* Synthesis, characterization and performance of polystyrene/PMMA blend membranes for potential water treatment. *Desalination* **431**, 35-46 (2018).
27. Mashayekhi F, *et al.* Adhesion Optimization between Incompatible Polymers through Interfacial Engineering. *Polymers (Basel)* **13**, 4273 (2021).
28. Gerthoffer A, *et al.* CIGS solar cells on ultra-thin glass substrates: Determination of mechanical properties by nanoindentation and application to bending-induced strain calculation. *Solar Energy Materials and Solar Cells* **166**, 254-261 (2017).
29. Kim H, *et al.* Direct coating of transparent and wear-resistant polysilsesquioxane on ultra-thin glass for flexible cover windows. *Progress in Organic Coatings* **187**, 108162 (2024).
30. Xie J, *et al.* Highly Foldable, Super-Sensitive, and Transparent Nanocellulose/Ceramic/Polymer Cover Windows for Flexible OLED Displays. *ACS Appl Mater Interfaces* **14**, 16658-16668 (2022).
31. Chen L, *et al.* Highly Transparent and Colorless Nanocellulose/Polyimide Substrates with Enhanced Thermal and Mechanical Properties for Flexible OLED Displays. *Advanced Materials Interfaces* **7**, 2000928 (2020).
32. Hao L, *et al.* Thickness dependence of structural, electrical and optical properties of indium tin oxide (ITO) films deposited on PET substrates. *Applied Surface Science* **254**, 3504-3508 (2008).
33. Vinogradov A, *et al.* Electro-mechanical properties of the piezoelectric polymer PVDF. *Ferroelectrics* **226**, 169-181 (1999).
34. Tian S, *et al.* Fabrication of a Transparent and Biodegradable Cellulose Film from Kraft Pulp via Cold Alkaline Swelling and Mechanical Blending. *ACS Sustainable Chemistry & Engineering* **10**, 10560-10569 (2022).
35. Johnston ID, *et al.* Mechanical characterization of bulk Sylgard 184 for microfluidics and microengineering. *Journal of Micromechanics and Microengineering* **24**, 035017 (2014).
36. Wang Y, *et al.* Study on mechanical properties, thermal stability and crystallization behavior of PET/MMT nanocomposites. *Composites Part B: Engineering* **37**, 399-407 (2006).
37. Carr JM, *et al.* Structure and transport properties of polyethylene terephthalate and poly(vinylidene fluoride-co-tetrafluoroethylene) multilayer films. *Polymer* **54**, 1679-1690 (2013).
38. Hogg A, *et al.* Ultra-thin layer packaging for implantable electronic devices. *Journal of Micromechanics and Microengineering* **23**, 075001 (2013).
39. Tsai M-H, *et al.* Transparent Polyimide Film with Improved Water and Oxygen Barrier Property by In-Situ Exfoliating Graphite *Advanced Engineering Materials* **18**, 582-590 (2016).
40. Wang C, *et al.* Effects of CF4 plasma treatment on the moisture uptake, diffusion, and WVTR of poly(ethylene terephthalate) flexible films. *Surface and Coatings Technology* **206**, 318-324 (2011).

41. Hsu PC, *et al.* A dual-mode textile for human body radiative heating and cooling. *Sci Adv* **3**, e1700895 (2017).
42. Plank NOV, *et al.* Facile synthesis of poly(methylsilsesquioxane) and MgO nanoparticle composite dielectrics. *Journal of Materials Research* **28**, 1490-1497 (2013).
43. Shi K, *et al.* Dielectric Modulated Cellulose Paper/PDMS-Based Triboelectric Nanogenerators for Wireless Transmission and Electropolymerization Applications. *Advanced Functional Materials* **30**, 1904536 (2020).
44. Zhou Y, *et al.* Flexible /PET/batio$_3$/ layer–layer composite film with enhanced dielectric properties fabricated by highly loaded /batio$_3$/ coating with acrylic resin as binder. *Journal of Applied Polymer Science* **132**, 42508 (2015).
45. Dallaev R, *et al.* Brief Review of PVDF Properties and Applications Potential. *Polymers* **14**, 4793 (2022).
46. Zhang N, *et al.* Light weight high temperature polymer film capacitors with dielectric loss lower than polypropylene. *Journal of Materials Science: Materials in Electronics* **26**, 9396-9401 (2015).
47. Shen J, *et al.* Stress relaxation of a soda lime silicate glass below the glass transition temperature. *Journal of Non-Crystalline Solids* **324**, 277-288 (2003).
48. Zalewski K, *et al.* A Review of Polysiloxanes in Terms of Their Application in Explosives. *Polymers* **13**, 1080 (2021).
49. Thomsen TB, *et al.* Influence of substrate crystallinity and glass transition temperature on enzymatic degradation of polyethylene terephthalate (PET). *New Biotechnology* **69**, 28-35 (2022).
50. Horibe H, *et al.* Effect of heat-treatment temperature after polymer melt and blending ratio on the crystalline structure of PVDF in a PVDF/PMMA blend. *Polymer Journal* **45**, 1195-1201 (2013).
51. Abdel-Hady EE, *et al.* Temperature effect on free volume of polymethylpentene studied by positron annihilation technique. *physica status solidi c* **6**, 2420-2422 (2009).
52. Szcześniak L, *et al.* Glass transition temperature and thermal decomposition of cellulose powder. *Cellulose* **15**, 445-451 (2008).
